# FAMILY DEMENTIA CAREGIVERS’ EPIPHANIES AND CHANGES IN THEIR APPROACH TO CARING FOLLOWING THE VIRTUAL DEMENTIA TOUR

**DOI:** 10.1093/geroni/igz038.2042

**Published:** 2019-11-08

**Authors:** Candace C Harrington, Candace C Harrington

**Affiliations:** 1 East Carolina University, Greenville, United States; 2 East Carolina University, Zebulon, North Carolina, United States

## Abstract

Previous interventional studies have failed to show long-term improvements in caregiver stress, health indices, burden, or delay in long-term care placement. The Virtual Dementia Tour® (VDT) provides a vicarious first-person perspective of symptoms related to dementia. This interpretative phenomenological study revealed family dementia caregivers’ perceptions of the VDT® and its impact on their perception of a person living with dementia. In-depth open semi-structured interviews were conducted with ten VDT® participants following a community event. Participants’ statements described a life-changing process with eye-opening epiphanies about the lived experience of dementia and served as a “call to action” to change their approach to caring. Innovative advances in family caregiving research are critical to support this valuable geriatric workforce. This original study provided new knowledge about the value of the VDT® to inform interventions that harness the unrecognized power of vicarious experiences like the VDT® for family dementia caregivers to improve long-term outcomes.

